# Semisupervised Learning Based Disease-Symptom and Symptom-Therapeutic Substance Relation Extraction from Biomedical Literature

**DOI:** 10.1155/2016/3594937

**Published:** 2016-10-16

**Authors:** Qinlin Feng, Yingyi Gui, Zhihao Yang, Lei Wang, Yuxia Li

**Affiliations:** ^1^College of Computer Science and Technology, Dalian University of Technology, Dalian 116024, China; ^2^School of Optoelectronics, Beijing Institute of Technology, Beijing 100081, China; ^3^Beijing Institute of Health Administration and Medical Information, Beijing 100850, China

## Abstract

With the rapid growth of biomedical literature, a large amount of knowledge about diseases, symptoms, and therapeutic substances hidden in the literature can be used for drug discovery and disease therapy. In this paper, we present a method of constructing two models for extracting the relations between the disease and symptom and symptom and therapeutic substance from biomedical texts, respectively. The former judges whether a disease causes a certain physiological phenomenon while the latter determines whether a substance relieves or eliminates a certain physiological phenomenon. These two kinds of relations can be further utilized to extract the relations between disease and therapeutic substance. In our method, first two training sets for extracting the relations between the disease-symptom and symptom-therapeutic substance are manually annotated and then two semisupervised learning algorithms, that is, Co-Training and Tri-Training, are applied to utilize the unlabeled data to boost the relation extraction performance. Experimental results show that exploiting the unlabeled data with both Co-Training and Tri-Training algorithms can enhance the performance effectively.

## 1. Introduction

In recent years, with the rapid growth of biomedical literature, the technology of information extraction (IE) has been extensively applied to relation extraction in this literature, for example, extracting the semantic relations between diseases, drugs, genes, proteins, and so forth [1–3]. The related challenges (e.g., BioCreative II protein-protein interaction (PPI) task [4], DDIExtraction 2011 [5], and DDIExtraction 2013 [6]) have been held successfully.

In our work, we focus on extracting the relations between diseases and their symptoms and symptoms and their therapeutic substances. These relations are defined the same as those in [4–6] and also annotated at the sentence level. The former is the relationship between a disease and its related physiological phenomenon in a sentence. For example, the sentence “many blood- and blood vessel-related characteristics are typical for* Raynaud* patients:* Blood viscosity* and* platelet aggregability* are high” shows that* blood viscosity* and* platelet aggregability* are physiological phenomenon of* Raynaud disease*. The latter is the relationship between a physiological phenomenon and the therapeutic substance that can relieve it in a sentence. For example, the sentence “*fish oil* and* its active ingredient eicosapentaenoic acid (EPA)* lowered* blood viscosity*” shows that* fish oil* and* EPA* can relieve the physiological phenomenon* (blood viscosity)*. These two kinds of relations can be further utilized to extract the relations between disease and therapeutic substance. As shown in the above example, it can be assumed that* fish oil* and* EPA* may relieve or heal* Raynaud disease*. Therefore, such information is important for drug discovery and disease treatment. Currently, a large amount of knowledge on diseases, symptoms, and therapeutic substances remains hidden in the literature and needs to be mined with IE technology.

Generally, the methods of extracting the semantic relation between biomedical entities include cooccurrence-based methods [7], pattern-based methods [8], and machine learning methods [9]. Cooccurrence-based methods use frequent cooccurrence to extract the relations between entities. This method is simple and shows very low precision for high recall [10]. Yen et al. developed a cooccurrence approach based on an information retrieval principle to extract gene-disease relationships from text [11]. Pattern-based methods define a series of patterns in advance and use pattern matching to extract the relations between entities. Huang et al. used a dynamic programming algorithm to compute distinguishing patterns by aligning relevant sentences and key verbs that describe protein interactions [12]. Since templates are manually defined, its generalization ability is not satisfactory. Machine learning methods, the most popular ones, use classification algorithms to extract the relations between entities from literature, such as support vector machine (SVM) [13], maximum entropy [14], and Naive Bayes [15]. Among others, kernel-based methods are widely used in relation extraction. These methods define different kernel functions to extract the relations between entities, such as graph kernel [16], tree kernel [17], and walk path kernel [18].

The machine learning methods belong to the supervised learning ones which need a large of labeled examples to train the model. However, currently no corpuses for extraction of disease-symptom and symptom-therapeutic substance relations are available. In addition, even if limited labeled data are available, it is still difficult to achieve satisfactory generalization ability for a classifier. To solve the problem, we first manually annotated two training sets for extracting the relations between the disease-symptom and symptom-therapeutic substance and then introduced the semisupervised learning methods to utilize the unlabeled data for training the models.

Semisupervised learning methods attempt to exploit the unlabeled data to help improve the generalization ability of the classifier with limited labeled data. They can be roughly divided into four categories, that is, generative parametric models [19], semisupervised support vector machines (S3VMs) [20], graph-based approaches [21], and Co-Training [22–27]. Co-Training was proposed by Blum and Mitchell [22]. This method requires two sufficient and redundant views which do not exist in most real-world scenarios. In order to relax this constraint, Zhou and Li proposed a Tri-Training algorithm that neither requires the instance space to be described with sufficient and redundant views nor puts any constraints on the supervised learning method [28]. The algorithm uses three classifiers, which can not only tackle the problem of determining how to label the unlabeled data, but also improve generalization ability of a classifier with unlabeled data. Wang et al. made a large number of studies on Co-Training and proved that if two views have large diversity, Co-Training is able to improve the learning performance by exploiting the unlabeled data even with insufficient views [23–25]. Until now, Tri-Training and Co-Training have been widely used in natural language processing. Pierce and Cardie [26] applied Co-Training to noun phrase recognition. They regarded the current word and the *k* words which appear before the current word in the document as a view and the *k* words appear after the current word as another view and then trained the classifiers on these two views with Co-Training algorithm. Mavroeidis et al. [29] applied Tri-Training algorithm to spam detection filtering and achieved a satisfactory result.

Meanwhile, the ensemble learning methods have been proposed, which combine the outputs of several base learners to form an integrated output for enhancing the classification performance. There are three popular ensemble methods, that is, Bagging [30], Boosting [31], and Random Subspace [32]. The Bagging method uses random independent bootstrap replicates from a training dataset to construct base learners and calculates the final result by a simple vote [30]. For Boosting method, the base learners are constructed on weighted versions of training set, which are dependent on previous base learners' results and the final result is calculated by a simple vote or a weighted vote [31]. The Random Subspace method uses random subspaces of the feature space to construct the base learners [32].

In our method, we regard three kernels (i.e., the feature kernel, graph kernel, and tree kernel which will be introduced in the following section) as three different views. Co-Training and Tri-Training algorithms are then employed to exploit the unlabeled data with these views and build the disease-symptom model and symptom-therapeutic substance model. Meanwhile, in the Tri-Training process, we adopted the ensemble learning method to integrate three individual kernels and achieved a satisfactory result.

## 2. Methods

### 2.1. Feature Kernel

The core work of the feature-based method is feature selection which has a significant impact on the performance. The following features are used in our feature-based kernel.

*(1) Word Feature*. Word feature uses two disordered sets of words which are between two concept entities (diseases, symptoms, and therapeutic substances) and surrounding two conceptual entities as the eigenvector. The features surrounding two concept entities' names include the left* M* words of the first concept entity name and the right* M* words of the second concept entity name (in our experiments,* M* is set to 4). 

*(2) N-Gram Word Feature*. In our method, we use* N*-gram (*N* = 1, 2, and 3 in our experiments) words from the left four words of the first concept entity to the right four words of the second concept as features.* N*-gram features enrich the word feature and add contextual information, which can effectively express the relation of concept entities. 

*(3) Position Feature*. The relative position information of word feature and* N*-gram feature for the concept entities has an important influence on relation extraction and, therefore, is introduced into our method. For example, “E1_L_feature” denotes a word feature or* N*-gram feature appears in the left of first concept entity; “E_B_feature” between two concept entities; “E2_R_feature” in the right of second concept entity. 

*(4) Interaction Word and Distance Features*. Some words such as “induce,” “action,” and “improve” often imply the existence of relations. Therefore, the existence of these words (we called interaction words) is chosen as a binary feature. In addition, we found that the shorter the distance between two concept entities is, the more likely the two concept entities have an interactive relationship. Therefore, the distance is chosen as a feature. For example, “DISLessThanTree” is a feature value showing that the distance between the two concept entities is less than three.

The initial eigenvector extracted with our feature-based kernel has a high dimension and includes many sparse features. In order to reduce the dimension, we employed the document frequency method [33] to select features. Initially, the feature-based kernel method extracts 248,000 features from the disease-symptom training set and we preserved the features with document frequencies exceeding five (a total of 12,000 features). Similarly, 345,000 features were extracted from the symptom-therapeutic substance training set and 13,700 features were retained.

### 2.2. Convolution Tree Kernel

In our method, convolution tree kernel *K*_*c*_(*T*_1_, *T*_2_), a special convolution kernel, is used to obtain useful structural information from substructure. It calculates the syntactic structure similarity between two parse trees by counting the number of common subtrees of the two parse trees rooted by *T*_1_ and *T*_2_:

(1) $$\begin{array}{c} K_{c}\left(\;T_{1},T_{2}\;\right)=\underset{n_{1}\in N_{1},n_{2}\in N_{2}}{\sum }\Delta \left(\;n_{1},n_{2}\;\right), \end{array}$$

where *N*_*j*_ denotes the set of nodes in the tree *T*_*j*_ and Δ(*n*_1_, *n*_2_) denotes the number of common subtrees of the two parse trees rooted by *n*_1_ and *n*_2_.

#### 2.2.1. Tree Pruning in Convolution Kernel

In our method, Stanford parser [34] is used to parse the sentences. Before a sentence is parsed, the concept entity pairs in the sentence are replaced with “ENTRY1” and “ENTRY2,” and other entities are replaced with “ENTRY.” Take gene-gene interaction between C0021764 and interleukin increases C0002395 risk (the sentence is processed with MetaMap, and the two concept entities are represented with their CUIs) for example. It is replaced with “gene-gene interaction between ENTRY1 and interleukin increases ENTRY2 risk.” Then, we use Stanford parser to parse the sentence to get a Complete Tree (CT). Since a CT includes too much contextual information which may introduce many noisy features, we used the method described in [35] to obtain the shortest path enclosed tree (SPT),and replace the CT with it. SPT is the smallest common subtree including the two concept entities, which is a part of CT.

#### 2.2.2. Predicate Argument Path

**Figure 1:**
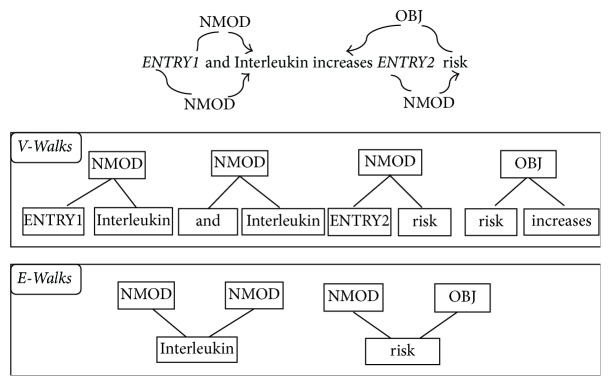
An example of a dependency graph. The candidate interaction pair is marked as “ENTRY1” and “ENTRY2.”

The representation of a predicate argument is a graphic structure, which expresses the deep syntactic and semantic relations between words. In the predicate argument structure, different substructures on the shortest path between the two concept entities have different information. An example of a dependency graph is shown in Figure 1. In our method, v-walk and e-walk features (which are both on the shortest dependency paths) are added into the tree kernel. V-walk contains the syntactic and semantic relations between two words. For example, in Figure 1, the relation between “ENTRY1” and “interleukin” is “NMOD” and the relation between “risk” and “increases” is “OBJ,” and so forth. E-walk contains the relations between a word and its two adjacent nodes. Figure 1 shows the relation of “interleukin” with its two adjacent nodes “NMOD” and “NMOD” and the relation of “risk” with its two adjacent nodes “NMOD” and “OBJ.”

### 2.3. Graph Kernel

**Figure 2:**
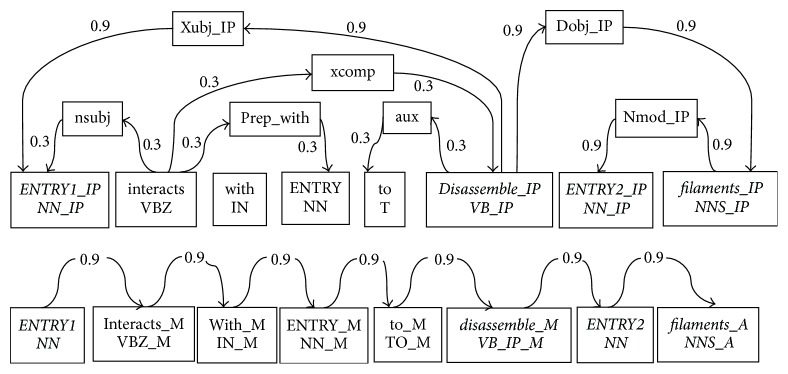
Graph kernel with two directed subgraphs. The candidate interaction pair is marked as “ENTRY1” and “ENTRY2.” In the dependency based subgraph all nodes in a shortest path are specialized using a post-tag (IP). In the linear order subgraph possible tags are (B)efore, (M)iddle, and (A)fter.

The graph kernel method uses the syntax tree to express a graph structure of a sentence. The similarity of two graphs is calculated by comparing the relation between two public nodes (vertices). Our method uses the all-paths graph kernel proposed by Airola et al. [16]. The kernel consists of two directed subgraphs, that is, a parse graph and a graph representing the linear order of words. In Figure 2 the upper part is the analysis of the structure subgraph and the lower part is the linear order subgraph. These two subgraphs denote the dependency structure and linear sequence of a sentence, respectively.

In our method, a simple weight allocation strategy is chosen; that is, the edges of the shortest path are assigned a weight of 0.9; other edges 0.3; all edges in the linear order subgraph 0.9. The representation thus allows us to emphasize the shortest path without completely disregarding potentially relevant words outside of the path. A graph kernel calculates the similarity between two input graphs by comparing the relations between common vertices (nodes). A graph matrix *G* is calculated as

(2) $$\begin{array}{c} G=L\overset{\infty }{\underset{n=1}{\sum }}A^{n}L^{T}, \end{array}$$

where *A* is an edge matrix whose rows and columns are indexed vertices. *A*_*ij*_ is a weight if edge *V*_*i*_ is connected to edge *V*_*j*_. *L* is the label matrix whose row indicates the label and column indicates the vertex. *L*_*ij*_ = 1 indicates that vertex *V*_*j*_ contains *i*th label. The graph kernel *K*(*G*, *G*′) is defined by using two input graph matrices *G* and *G*′ [15].

(3) $$\begin{array}{c} K\left(\;G,G^{\prime }\;\right)=\overset{L}{\underset{i=1}{\sum }}\;\overset{L}{\underset{j=1}{\sum }}G_{ij}G_{ij}^{\prime } \end{array}$$

### 2.4. Co-Training Algorithm

The initial Co-Training algorithm (or standard Co-Training algorithm) was proposed by Blum and Mitchell [22]. They assumed that the training set has two sufficient and redundant views; namely, the set of attributes meets two conditions. First, each attribute set is sufficient to describe the problem; that is, if the training set is sufficient, each attribute set is able to learn a strong classifier. Second, each attribute set is conditionally independent of the other given the class label. Our Co-Training algorithm is described in Algorithm 1:

**Algorithm 1 (Co-Training algorithm).**

(1) Input is as follows:
 The labeled data *L* and the unlabeled data *U*
 Initialize training set *L*_1_, *L*_2_ (*L*_1_ = *L*_2_ = *L*)
 Sufficient and redundant views: *V*_1_, *V*_2_
 Iteration number:* N*
(2) Process is as follows:
  (2.1) Create a pool *u* of examples by choosing *n* examples at random from *U*, *U* = *U* − *u*.
  (2.2) Use *L*_1_ to train a classifier *h*_1_ in *V*_1_.
   Use *L*_2_ to train a classifier *h*_2_ in *V*_2_.
  (2.3) Use *h*_1_ and *h*_2_ to label the examples from* u*.
  (2.4) Take *m* positive examples and *m* negative examples out, which were consistently labeled by *h*_1_ and *h*_2_. Then take *p* positive examples out from the *m* positive examples and add them to *L*_1_ and *L*_2_, respectively. Choose 2*m* examples from *U* to replenish* u*, *U* = *U* − 2*m*, *N* = *N* − 1.
  (2.5) Repeat the processes (2.2)–(2.4) until the unlabeled corpora *U* are empty or the number of unlabeled data in *u* is less than a certain number or *N* = 0.
(3) Outputs are as follows:
 The classifiers *h*_1_ and *h*_2_

### 2.5. Tri-Training Algorithm

The Co-Training algorithm requires two sufficient and redundant views. However, this constraint does not exist in most real-world scenarios. The Tri-Training algorithm neither requires the instance space to be described with sufficient and redundant views and nor puts any constraints on the supervised learning algorithm [28]. In this algorithm, three classifiers are used, which can tackle the problem of determining how to label the unlabeled data and produce the final hypothesis. Our Tri-Training algorithm is described in Algorithm 2.

In addition, the different classifiers calculate the similarity with different aspects between the two sentences. Combining the similarities can reduce the danger of missing important features. Therefore, in each Tri-Training round, two different ensemble strategies are used to integrate the three classifiers for further performance improvement. The first strategy integrates the classifiers with a simple voting method. The second strategy assigns each classifier with a different weight. Then the normalized output *K* of three classifier outputs *K*_*m*_ (*m* = 1,2, 3) is defined as

(4) K=∑m=1MσmKm∑m=1Mσm=1,σm≥0,  ∀m,

where *M* represents the number of classifiers (*M* = 3 in our method).

**Algorithm 2 (Tri-Training algorithm).**

(1) Input is as follows:
 The labeled data* L* and the unlabeled data* U*
 Initializing training set *L*_1_, *L*_2_, *L*_3_ (*L*_1_ = *L*_2_ = *L*_3_ = *L*)
 Selecting views: *V*_1_, *V*_2_, and *V*_3_
 Iterations number:* N*
(2) Process is as follows:
  (2.1) Create a pool *u* of examples by choosing *n* examples at random from *U*, *U* = *U* − *u*.
  (2.2) Use *L*_1_ to train a classifier *h*_1_ in *V*_1_.
   Use *L*_2_ to train a classifier *h*_2_ in *V*_2_.
   Use *L*_3_ to train a classifier *h*_3_ in *V*_3_.
  (2.3) Use *h*_1_, *h*_2_, and *h*_3_ to label examples from *u*.
  (2.4) Take *m* positive examples and *m* negative examples out, which were consistently labeled by *h*_1_, *h*_2_, and *h*_3_. Then take *p*_1_ positive examples from the *m* positive examples and add them to *L*_1_, *L*_2_, and *L*_3_, respectively; take *p*_2_ negative examples from the *m* negative examples and add them to *L*_1_, *L*_2_, and *L*_3_, respectively. Choose 2*m* examples from *U* to replenish *u*, *U* = *U* − 2*m*, *N* = *N* − 1.
  (2.5) Repeat the processes (2.2)–(2.4) until the unlabeled corpora *U* are empty or the number of unlabeled data in *u* is less than a certain number or *N* = 0.
(3) Outputs are as follows:
 The classifiers *h*_1_, *h*_2_, and *h*_3_

## 3. Experiments and Results

### 3.1. Experimental Datasets

In our experiments, the disease and symptom corpus data was obtained through searching Semantic MEDLINE Database [36] using 200 concepts chosen from MeSH (Medical Subject Headings) with semantic type “*Disease or Syndrome.”* Since these sentences (corpus data) have been processed by SemRep [37], a natural language processing tool based on the rule to identify relationship in the MEDLINE documents, the possibility of the relation between the two concept entities in the sentences is high. To limit the semantic types of two concept entities in a sentence, we only preserved the sentences containing the concepts of the needed semantic types (i.e.,* biologic function, cell function, finding, molecular function, organism function, organ or tissue function, pathologic function, phenomenon or process*, and* physiologic function*). Finally, we obtained a total of about 20,400 sentences from which we manually constructed two labeled datasets as the initial training set *T*_initial_ (598 labeled sentences as shown in Table 1) and test set (499 labeled sentences), respectively.

During the manual annotation, the following criteria are applied: the disease and symptom relationship indicates that the symptom is a physiological phenomenon of the disease. If an instance in a sentence semantically expresses the disease and symptom relationship, it is labeled as a positive example. As in the example provided in Section 1, the sentence “many blood- and blood vessel-related characteristics are typical for* Raynaud* patients:* blood viscosity* and* platelet aggregability* are high” contains two positive examples, that is,* Raynaud* and* blood viscosity* and* Raynaud* and* platelet aggregability*. In addition, some special relationships such as “B in A” and “A can change B” are also classified as the positive examples since they show a physiological phenomenon (B) occurs when someone has the disease (A). However, if a relation in a sentence is only a cooccurrence one, it is labeled as a negative example. For the patterns such as “A is a B” and “A and B” they are labeled as the negative examples since “A is a B” is a “IS A” relation and “A and B” is a coordination relation, which are not the relations we need.

The symptom-therapeutic substance corpus data was obtained as follows. First, some “Alzheimer's disease” related symptom terms were obtained from the Semantic MEDLINE Database. Then these symptom terms were used to search the database for the sentences which contain the query terms and terms belonging to the semantic types of therapeutic substance (e.g.,* pharmacologic substance* and* organic chemical*). We obtained about 20,500 sentences and then manually annotated about 1,100 sentences as the disease-symptom corpora: 600 labeled sentences are used as the initial training set and the remaining 498 labeled sentences as the test set. Similar to the disease and symptom relationship annotation, the following criteria are applied: the symptom-therapeutic substance relationship indicates that a therapeutic substance can relieve a physiological phenomenon. If an instance in a sentence semantically expresses the symptom-therapeutic substance relationship, it is labeled as a positive example. As in the example provided in Section 1, the sentence “*fish oil* and* its active ingredient eicosapentaenoic acid (EPA)* lowered* blood viscosity*” contains two positive examples, that is,* fish oil* and* blood viscosity* and* EPA* and* blood viscosity*.

When the manual annotation process was completed, the level of agreement was estimated. Cohen's kappa scores between each annotator of two corpora are 0.866 and 0.903, respectively, and content analysis researchers generally think of a Cohen's kappa score more than 0.8 as good reliability [38]. In addition, the two corpora are available for academic use (see Supplementary Material available online at http://dx.doi.org/10.1155/2016/3594937).

### 3.2. Experimental Evaluation

The evaluation metrics used in our experiments are precision (*P*), recall (*R*),* F*-score (*F*), and Area under Roc Curve (AUC) [39]. They are defined as follows:

(5) P=TPTP+FP

(6) R=TPTP+FN

(7) F=2∗P∗RP+R

(8) AUC=∑i=1m+∑j=1m−Hxi−yjm+m−,

where TP denotes true interaction pair; TN denotes true noninteraction pair; FP denotes false interaction pair; and FN denotes false noninteraction pair.* F*-score is the balanced measure for quantifying the performance of the systems. In addition, the AUC is also used to evaluate the performance of our method. It is not affected by the distribution of data, and it has been advocated to be used for performance evaluation in the machine learning community [40]. In formula (8), *m*_+_ and *m*_−_ are the numbers of positive and negative examples, respectively, and *x*_1_,…, *x*_*m*_+__ are the outputs of the system for the positive examples, and *y*_1_,…, *y*_*m*_−__ are the ones for the negative examples. The function *H*(*r*) is defined as follows:

(9) $$\begin{array}{c} H\left(\;r\;\right)=\left\{\begin{array}{cc} 1, & r>0\\ 0.5, & r=0\\ 0, & r<0. \end{array}\right. \end{array}$$

### 3.3. The Initial Performance of the Disease-Symptom Model

Table 2 shows the performance of the classifiers on the initial disease-symptom test set. Feature kernel and graph kernel achieve almost the same performance which is better than that of tree kernel. When the three classifiers are integrated with the same weight, the higher* F*-score (75.00%) is obtained while, when they are integrated with a weight ratio of 4 : 4 : 2, the* F*-score is a bit lower than that of feature kernel. However, in both cases, the AUC performances are improved, which shows that since different classifiers calculate the similarity with different aspects between two sentences, combining these similarities can boost the performance.

#### 3.3.1. The Performance of Co-Training on the Disease-Symptom Test Set

**Figure 3:**
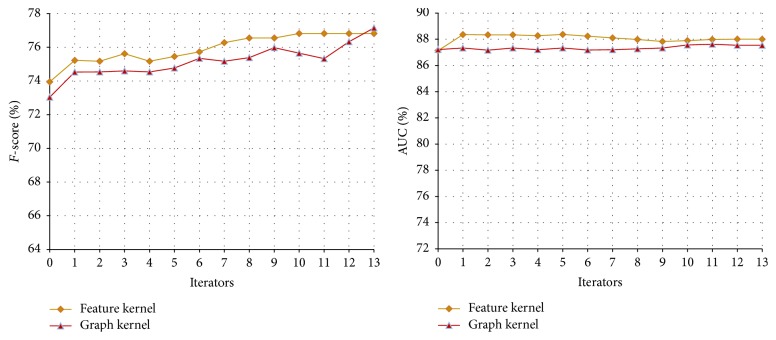
Co-Training performance curve of feature kernel and graph kernel on the disease-symptom test set.

**Figure 4:**
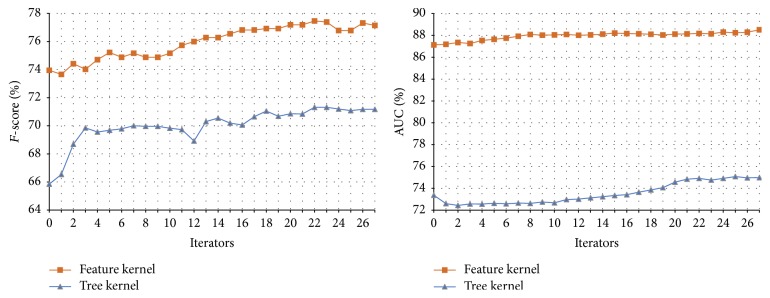
Co-Training performance curve of feature kernel and tree kernel on the disease-symptom test set.

**Figure 5:**
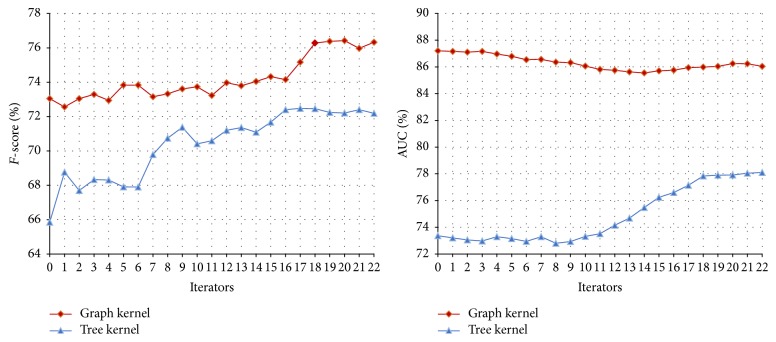
Co-Training performance curve of graph kernel and tree kernel on the disease-symptom test set.

In our method, the feature set for the disease-symptom model is divided into three views: the feature kernel, graph kernel, and tree kernel. In Co-Training experiments, to compare the results of each combination of two views, the experiments are divided into three groups as shown in Table 3. Each group uses same experimental parameters; that is,* u* = 4,000,* m* = 300, and* p* = 100 (*u*,* m,* and *p* in Algorithm 1). The performance curves of different combinations are shown in Figures 3, 4, and 5, respectively, and their final results with different iteration times (13, 27 and 22, resp.) are shown in Table 3.

From Figures 3, 4, and 5, we can obtain the following observations. (1) With the increase of the iteration time and more unlabeled data added to the training set, the* F*-score shows a rising trend. The reason is that, as the Co-Training process proceeds, more and more unlabeled data are labelled by one classifier for the other, which improves the performance of both classifiers. However, after a number of iterations, the performance of the classifiers could not be improved any more since too much noise (false positives and false negatives) may be introduced from the unlabeled data. (2) The AUC of classifiers have different trends with different combinations of the views. The AUC of the feature kernel fluctuate around 88% while the ones of the graph kernel fluctuate between 85% and 87%. In contrast, all of the tree kernel's AUC have a rising trend since the performance of the initial tree kernel classifier is relatively low and then improved with the relatively accurate labelled data provided by feature kernel or graph kernel.

In fact, the performance of semisupervised learning algorithms is usually not stable because the unlabeled examples may often be wrongly labeled during the learning process [28]. At the beginning of the Co-Training, the number of the noises is limited and unlabeled data added to the training set can help the classifiers improve the performance. However, after a number of learning rounds, more and more noises introduced will cause the performance decline.

#### 3.3.2. The Performance of Tri-Training on the Disease-Symptom Test Set

**Figure 6:**
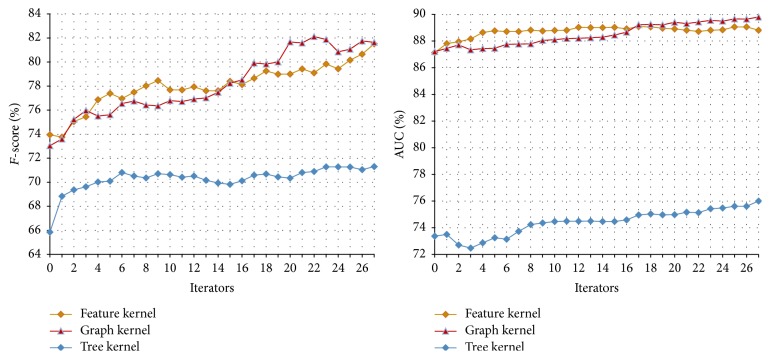
The Tri-Training performance on the disease-symptom test set.

In our method, we select three views to conduct the Tri-Training, that is, the feature kernel, graph kernel, and tree kernel. In each Tri-Training round, SVM is used to train the classifier on each view. The parameters are set as follows:* u* = 4,000,* m* = 300, *p*_1_ = 100, *p*_2_ = 0, and *N* = 27 (*u*,* m*, *p*_1_, *p*_2_, and *N* in Algorithm 2). Here *p*_2_ = 0 means that only the positive examples are added into the training set. In this way, the recall of the classifier can be improved (the recall is defined as the number of true positives divided by the total number of examples that actually belong to the positive class and usually more positive examples in the training set will improve the recall) since it is lower compared with the precision (see Table 2). The results are shown in Table 4 and Figure 6.

Compared with the performances of the classifiers on the initial disease-symptom test set shown in Table 2, the ones achieved through Tri-Training are significantly improved. This shows that Tri-Training can exploit the unlabeled data and improve the performance more effectively. The reason is that, as mentioned in Section 1, the Tri-Training algorithm can achieve satisfactory results while neither requiring the instance space to be described with sufficient and redundant views nor putting any constraints on the supervised learning method.

In addition, when three classifiers are integrated either with the same weight or with a weight ratio of 4 : 4 : 2, the higher* F*-scores and AUCs are obtained. Furthermore, comparing the performance of Co-Training and Tri-Training shown in Tables 3 and 4, we found that, in most cases, Tri-Training outperforms Co-Training. The reason is that, through employing three classifiers, Tri-Training is facilitated with good efficiency and generalization ability because it could gracefully choose examples to label and use multiple classifiers to compose the final hypothesis [28].

### 3.4. The Performance of the Symptom and Therapeutic Substance Model

Table 5 shows the performances of the classifiers on the initial symptom-therapeutic substance test set. Similar to the results on the initial disease-symptom test set, the feature kernel achieves the best performance while the tree kernel performs the worst. One difference is that when the three classifiers are integrated with a weight ratio of 4 : 4 : 2, the higher* F*-score and AUC are obtained while, when they are integrated with the same weight, the* F*-score and AUC are a little lower than those of feature kernel.

#### 3.4.1. The Performance of Co-Training on the Symptom and Therapeutic Substance Test Set

**Figure 7:**
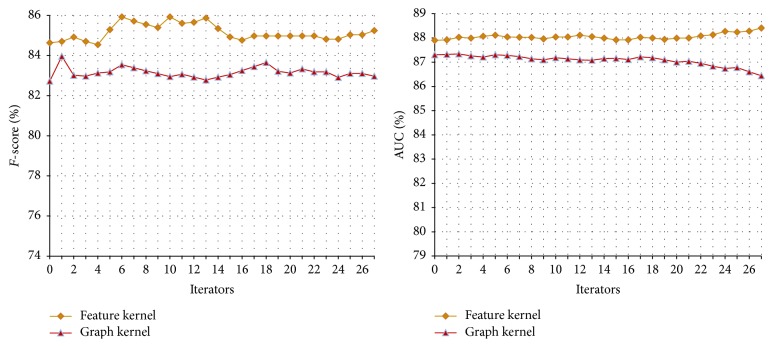
Co-Training performance curve of feature kernel and graph kernel on the symptom-therapeutic substance test set.

**Figure 8:**
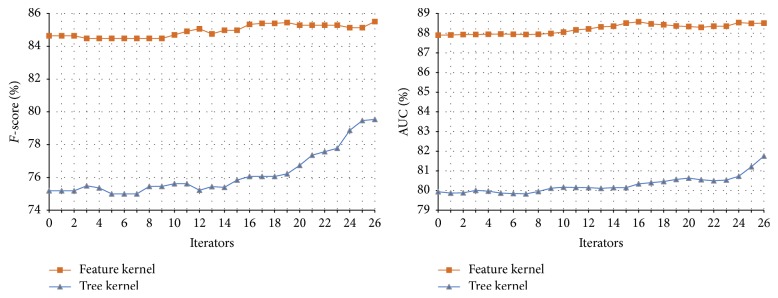
Co-Training performance curve of feature kernel and tree kernel on the symptom-therapeutic substance test set.

**Figure 9:**
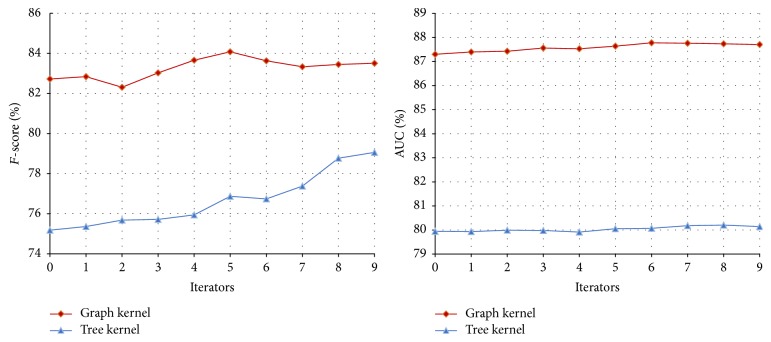
Co-Training performance curve of graph kernel and tree kernel on the symptom-therapeutic substance test set.

Similar to that in the disease-symptom experiments, the feature set for the symptom-therapeutic substance model is also divided into three views: the feature, graph, and tree kernels. The experiments are divided into three groups. Each group uses the same experimental parameters; that is,* u* = 4,000,* m* = 300, and* p* = 100. The performance curves of different combinations are shown in Figures 7, 8, and 9 and their final results with different iteration times (27, 26, and 9, resp.) are shown in Table 6.

From the figures, we can draw similar conclusions as from the disease-symptom experiments. In most cases, the performance can be improved through the Co-Training process while they are usually not stable since noise will be introduced during the learning process.

#### 3.4.2. The Performance of Tri-Training on the Symptom and Therapeutic Substance Test Set

**Figure 10:**
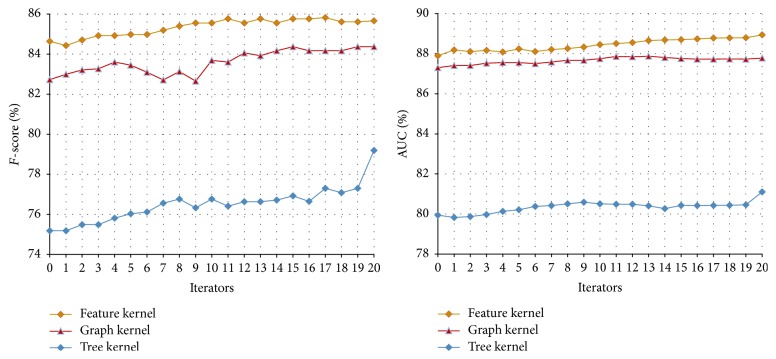
The results of Tri-Training on the symptom-therapeutic substance test set.

In the experiments of Tri-Training on the symptom-therapeutic substance, the parameters are set as follows:* u* = 4,000,* m* = 300, *p*_1_ = 100, *p*_2_ = 0, and *N* = 27 (*u*,* m*, *p*_1_, *p*_2_, and *N* in Algorithm 2). The results are shown in Table 7 and Figure 10.

Compared with the performance of the classifiers on the initial symptom-therapeutic substance test set shown in Table 6, the ones achieved through Tri-Training are also improved as in the disease-symptom experiments. This verifies that the Tri-Training algorithm is effective in utilizing the unlabeled data to boost the relation extraction performance once again. When the three classifiers are integrated with a weight ratio of 4 : 4 : 2, a better AUC is obtained.

Comparing the performance of Co-Training and Tri-Training on the symptom-therapeutic substance test set as shown in Tables 6 and 7, we found that, in most cases, Tri-Training outperforms Co-Training, which is consistent with the results achieved in the disease-symptom experiments. This is due to the better efficiency and generalization ability of Tri-Training over Co-Training.

In addition, the performances of the classifiers on the disease-symptom corpus are improved more than those on the symptom-therapeutic substance corpus. There are two reasons for that. First, on the symptom-therapeutic substance corpus, the classifiers have better performance. Therefore, the Co-training and Tri-training algorithms have less room for the performance improvement. Second, as the Co-training and Tri-training process proceeds, more unlabeled data are added into the training set, which could introduce new information for the classifiers. Therefore, the recalls of the classifiers are improved. Meanwhile, more noise is also introduced causing the precision decline. For the initial classifiers, the higher the precision is, the less the noise is introduced in the iterative process, and the performance of the classifier would be improved. As a summary, if the initial classifiers have big difference, the performance can be improved through two algorithms. In the experiment, when more unlabeled data are added to the training set, the difference between the classifiers becomes smaller. Thus, after a number of iterations, performance could not be improved any more.

### 3.5. Some Examples for Disease-Symptom and Symptom-Therapeutic Substance Relations Extracted from Biomedical Literatures

Some examples for disease-symptom or symptom-therapeutic substance relations extracted from biomedical literatures are shown in Tables 8 and 9.  Table 8 shows some symptoms of disease C0020541 (*portal hypertension*). One sentence containing the relation between* portal hypertension* and its symptom C0028778 (*block*) is provided.  Table 9 shows some relations between the symptom C0028778 (*block*) and some therapeutic substances, in which the sentences containing the relations are provided.

## 4. Conclusions and Future Work

Models for extracting the relations between the disease-symptom and symptom-therapeutic substance are important for further extracting knowledge about diseases and their potential therapeutic substances. However, currently there is no corpus available to train such models. To solve the problem, we first manually annotated two training sets for extracting the relations. Then two semisupervised learning algorithms, that is, Co-Training and Tri-Training, are applied to explore the unlabeled data to boost the performance. Experimental results show that exploiting the unlabeled data with both Co-Training and Tri-Training algorithms can enhance the performance. In particular, through employing three classifiers, Tri-training is facilitated with good efficiency and generalization ability since it could gracefully choose examples to label and use multiple classifiers to compose the final hypothesis [28]. In addition, its applicability is wide because it neither requires sufficient and redundant views nor puts any constraint on the employed supervised learning algorithm.

In the future work, we will study more effective semisupervised learning methods to exploit the numerous unlabeled data pieces in the biomedical literature. On the other hand, we will apply the disease-symptom and symptom-therapeutic substance models to extract the relations between diseases and therapeutic substances from biomedical literature and predict the potential therapeutic substances for certain diseases [41].

## Supplementary Material

The Supplementary Material is our manually annotated corpus of disease and symptom and symptom and therapeutic substance.

## Figures and Tables

**Table 1:** The details of two corpora.

Corpus	Training set		Test set		Unlabeled data
	Positive	Negative	Positive	Negative	Total
Diseases and symptoms	299	299	249	250	19,298
Symptoms and therapeutic substances	300	300	249	249	19,392

**Table 2:** The initial results on the disease-symptom test set. Method 1 integrates three classifiers (the feature kernel, graph kernel, and tree kernel) with the same weight while Method 2 integrates them with a weight ratio of 4 : 4 : 2.

Method	*P*	*R*	*F*-score	AUC
Feature kernel	91.38	62.11	73.95	87.13
Graph kernel	93.87	59.77	73.04	87.21
Tree kernel	69.10	62.89	65.85	73.37
Method 1	92.05	63.28	**75.00**	89.47
Method 2	92.81	60.55	73.29	**89.74**

**Table 3:** The results obtained with Co-Training on the disease-symptom test set. Combination method integrates three classifiers (the feature kernel, graph kernel, and tree kernel) with the same weight.

Combination	View	*P*	*R*	*F*-score	AUC
Feature and graph kernel	Feature kernel	88.32	67.97	76.82	88.01
	Graph kernel	**83.26**	71.88	77.15	87.54
	Combination	74.91	85.16	79.71	**88.66**

Feature and tree kernel	Feature kernel	86.06	69.92	77.15	88.51
	Tree kernel	57.80	92.58	71.17	74.99
	Combination	75.08	87.11	**80.65**	87.18

Graph and tree kernel	Graph kernel	84.04	69.92	76.33	86.04
	Tree kernel	58.10	95.31	72.19	78.10
	Combination	82.43	76.95	79.60	86.84

**Table 4:** The results obtained with Tri-Training on the disease-symptom test set. Method 1 integrates three classifiers (the feature kernel, graph kernel, and tree kernel) with the same weight while Method 2 integrates them with a weight ratio of 4 : 4 : 2.

Method	*P*	*R*	*F*-score	AUC
Feature kernel	83.00	80.08	81.51	88.80
Graph kernel	77.74	85.94	81.63	89.80
Tree kernel	57.38	94.14	71.30	76.00
Method 1	79.79	87.89	**83.64**	**91.57**
Method 2	79.93	85.55	82.64	90.75

**Table 5:** The initial results on the symptom-therapeutic substance test set.

Method	*P*	*R*	*F*	AUC
Feature kernel	79.30	90.76	84.64	87.90
Graph kernel	76.27	90.36	82.72	87.30
Tree kernel	68.90	82.73	75.18	79.94
Method 1	75.99	92.77	83.54	87.59
Method 2	77.81	94.38	**85.30**	**88.94**

**Table 6:** The results obtained with Co-Training on the symptom-therapeutic substance test set.

Combination	View	*P*	*R*	*F*	AUC
Feature kernel and graph kernel	Feature kernel	78.00	93.98	85.25	88.41
	Graph kernel	71.51	98.80	82.97	86.44
	Combination	77.45	95.18	85.40	**89.10**

Feature kernel and tree kernel	Feature kernel	78.72	93.57	85.51	88.51
	Tree kernel	67.13	97.59	79.54	81.75
	Combination	77.51	96.79	**85.66**	88.61

Graph kernel and tree kernel	Graph kernel	74.14	95.58	83.51	87.71
	Tree kernel	67.82	94.78	79.06	80.14
	Combination	71.05	97.59	82.23	86.24

**Table 7:** The results of Tri-Training on symptom-therapeutic substance test set.

	*P*	*R*	*F*	AUC
Feature kernel	78.98	93.57	**85.66**	88.94
Graph kernel	74.31	97.59	84.37	87.78
Tree kernel	68.01	94.78	79.19	81.10
Method 1	74.77	98.80	85.12	88.08
Method 2	75.62	98.39	85.51	**89.13**

**Table 8:** Some disease-symptom relations extracted from biomedical literature.

Disease	Symptom	Sentence
C0020541 (portal hypertension)	C0028778 (block)	*C0020541* as C2825142 of intrahepatic *C0028778* accounted for 83% of the patients (C0023891 65%, meta-C0022346 12%) and C0018920 11%
	C1565860	
	C0035357	
	C0005775	
	C0014867	
	C0232338	

**Table 9:** Some symptom-therapeutic substance relations extracted from biomedical literature.

Symptom	Therapeutic substance	Sentence
C0028778 (block)	C0017302 (general anesthetic agents)	Use-dependent conduction *C0028778* produced by volatile *C0017302*
	C0006400 (bupivacaine)	Epidural ropivacaine is known to produce less motor *C0028778* compared to *C0006400* at anaesthetic concentrations
	C0053241 (benzoquinone)	In contrast, *C0053241* and hydroquinone led to g2-*C0028778* rather than to a mitotic arrest
